# Effects of timing of adjunctive systemic antibiotics on the clinical outcome of periodontal therapy: A systematic review 

**DOI:** 10.4317/jced.56324

**Published:** 2020-03-01

**Authors:** Lamiaa Abdallaoui-Maan, Amal Bouziane

**Affiliations:** 1 Department of periodontology, Faculty of Dental Medicine, Mohammed V University in Rabat, Morocco; 2 Laboratory of Biostatistics, Clinical Research and Epidemiology, Mohammed V University in Rabat, Morocco

## Abstract

**Background:**

Many systematic reviews and meta-analysis have indicated beneficial effects of adjunctive systemic antibiotics in periodontal therapy in specific situations. However, some essential issues such as the ideal time of their administration during periodontal therapy remain unanswered. This systematic review aimed to determine at which phase of periodontal treatment would adjunctive systemic antibiotics lead to the best clinical outcomes, during the active phase or in the reevaluation phase.

**Material and Methods:**

Searches in the databases Medline, Scopus and Cochrane Library were conducted. The randomized clinical trials and retrospective cohort studies comparing the clinical benefits of adjunctive systemic antibiotic administration in the active phase of periodontal treatment versus their administration in the reevaluation phase were included. The primary outcomes assessed were differences in clinical changes in periodontal pocket depth and clinical attachment loss at all post-treatment phases.

**Results:**

Of the 6209 records identified, two randomized clinical trials and two retrospective cohort studies were eligible according to inclusion criteria. Two studies suggested there were greater clinical benefits when systemic antibiotics were prescribed during the active phase of periodontal therapy than in the reevaluation phase while two other studies showed no significant difference in clinical outcomes at 6 months between these two different timing of administration.

**Conclusions:**

The evidence available and evaluated in this systematic review is of heterogeneous quality and limited by the restricted number of studies and their dissimilarities in their study design and outcome reporting. Despite insufficient evidence to determine the ideal time to the adjunctive systemic antibiotic administration in the periodontal therapy, it seems that prescription of systemic antibiotic at the active phase of periodontal therapy leads to better clinical outcomes.

** Key words:**Active phase; periodontal therapy, periodontitis, reevaluation, systemic antibiotics, timing.

## Introduction

The use of systemic antimicrobials as adjunct to mechanical therapy has been indicated to potentiate the effects of non-surgical mechanical therapy in the cases of aggressive periodontitis, severe and progressive periodontitis and in periodontitis associated with specific microbiological profiles (1,2). Many reports, systematic reviews and meta-analysis have indicated beneficial effects of systemic antibiotics for patients with periodontal disease in these clinical situations (1-5) with more pronounced effects in aggressive periodontitis than in chronic periodontitis and in initially deep sites compared with moderately deep sites (6). A wide range of systemic antibiotics has been tested in clinical studies demonstrating superiority of the combination of amoxicillin and metronidazole over any other antibiotic agent in the treatment of different forms of periodontitis. However, there is currently insufficient high quality evidence to recommend a specific agent, dose or duration (7). The optimal time of antimicrobial drug administration is another subject of discussion, as it remains controversial whether adjunctive systemic antibiotics should preferably be administered during the initial non-surgical phase, or during the subsequent re-treatment at reevaluation.

At present, there is very little data to guide the selection of the most appropriate timing of antibiotic administration (8). Basing on some biological concepts, some studies recommend the use of systemic adjunctive antibiotic agents at the initial phase of treatment (9-11), as it was recommended in the consensus report of the sixth European Workshop on Periodontology (12). However, in light of the enhanced antibiotic resistance development, other reports support that it would be more judicious to reevaluate each patient’s response to non-surgical periodontal therapy before deciding to prescribe or not adjunctive systemic antibiotic therapy (6).

Therefore, this systematic review aims to determine which is the optimal time of systemic antibiotic administration as an adjunct to periodontal therapy, during the active non-surgical phase or the reevaluation phase.

## Material and Methods

-Protocol and registration

This Systematic review was conducted according to the Preferred Reporting Items for Systematic Review and Meta-Analysis (PRISMA) statement (13) and registered at the International Prospective Register of Systematic Reviews (PROSPERO) under the number CRD42019123386.

-Focused Question

The review was conducted to assess whether timing of systemic adjunctive antibiotics influenced the outcomes of periodontal therapy. Specifically, the purpose of this review is to answer the following question: “Would adjunctive systemic antibiotic administration in the active phase of periodontal therapy provides greater clinical benefits than in the reevaluation phase?”

-Eligibility Criteria

Studies were assessed for eligibility based on the following criteria:

1. Study type: randomized control trials (RCT), or controlled clinical trials (CCT), and retrospective cohort studies (RCS) were eligible for inclusion. Case-control studies were excluded. A minimum follow-up period of two months was required, but no restrictions were placed on the type of setting, language or year of publication.

2. Participants: patients with clinically diagnosed periodontitis were eligible for inclusion. Patients who didn’t require antibiotic prescription were excluded.

3. Intervention: clinical studies comparing at least two groups, one test (systemic antibiotic administered at the reevaluation phase of periodontal therapy) and one control (systemic antibiotic administered at the active phase of periodontal therapy) were eligible for inclusion. The studies administering systemic antibiotic without associated mechanical debridement or using local antibiotics or antibiotics in sub-antimicrobial doses were excluded.

4. Outcome variables were differences in clinical changes in Periodontal Pocket Depth (PPD) and Clinical Attachment Loss (CAL) at all post-treatment points between the test and control groups.

-Information Sources

An electronic search of Medline, Scopus and the Cochrane Library databases was performed on the 26th of October 2018 for eligible clinical studies. The reference lists of all included studies and relevant reviews were manually cross-referenced to complete data collection.

-Search Strategy

The following search strategy was used for the Medline database and adapted adequately for the Scopus and Cochrane Library searches:

≠1 (“periodontal diseases” OR periodontitis OR “chronic periodontitis” OR “aggressive periodontitis” OR “periodontal infection”) AND (“anti-infective agents” OR “antibacterial agents” OR “antimicrobial” OR antibiotics) AND (tim* OR delay OR phase) NOT photodynamic.

-Study Selection

The search results were downloaded into a Zotero library. After removing duplicates, the library was saved and two reviewers (LA and AB) screened titles and abstracts for eligibility. Full text articles for all studies that appeared to meet the inclusion criteria or could not be excluded for sure were obtained. Each article was assessed independently against the eligibility criteria by two reviewers (LA and AB) and reasons for exclusion were reported. Disagreements between reviewers were argued and resolved by discussion.

-Data Collection & Items

Two independent reviewers (LA and AB) collected Data from each included article. The following items were recorded within each included study: sample characteristics, length of the study, diagnosis, treatment performed in test groups and controls (including the protocol of scaling and root planning (SRP) (full-mouth or by quadrant), the antibiotic molecule, it’s dose, timing, frequency and duration and follow-up. The comparison of primary and secondary outcomes between test and control groups with statistical significance were also recorded.

-Assessment of Risk of bias 

Two independent review authors (LA and AB) assessed the methodological quality of each study. Discrepancies were resolved by discussion to reach a consensus.

Assessment of Risk of bias in clinical trials included was performed in accordance with the Cochrane Handbook for Systematic Reviews of Interventions (14). Studies were assessed on seven criteria constituting potential sources of bias: Sequence generation, Allocation concealment, Blinding of participants and personnel, Blinding of outcome assessors, Incomplete outcome data, Selective outcome reporting, Others bias. Each criterion was judged at low risk of bias(yes), high risk of bias (no) or unclearly determined (unclear). The risk of bias of each study was then assessed as: “Low risk of bias”: if “yes” is found in all domains; “High Risk of Bias”: if “no” is met in any of the domains and “Unclear Risk of Bias”: if “unclear” is recorded in any of the domains.

Assessment of Risk of bias in retrospective cohort studies included was performed in accordance with the Newcastle-Ottawa quality assessment scale for cohort studies (15). This scale is a ‘star’ rating system comprising three domains: selection of study groups, comparability of groups, and outcome of interest assessment. Each criterion fulfilled is awarded by a one star.

A study can be given a maximum of one star for each numbered criteria within the Selection and Outcome categories. A maximum of two stars can be allocated for Comparability category. A maximum of nine stars can be given for each study. The following thresholds were used for converting the Newcastle-Ottawa scales to Agency for Healthcare Research and Quality (AHRQ) standards (good, fair and poor) (15):

• If 3 or 4 stars were allocated in selection domain AND one or 2 stars in comparability domain AND 2 or 3 stars in outcome/exposure domain, the RCS is considered of “good quality”.

• If 2 stars were allocated in selection domain AND one or 2 stars in comparability domain AND 2 or 3 stars in outcome/exposure domain, the RCS is considered of “fair quality”.

• If no or one star was allocated in selection domain OR no star in comparability domain or one star in outcome/exposure domain, the RCS is considered of “poor quality”.

## Results

-Study selection

**Figure 1:**
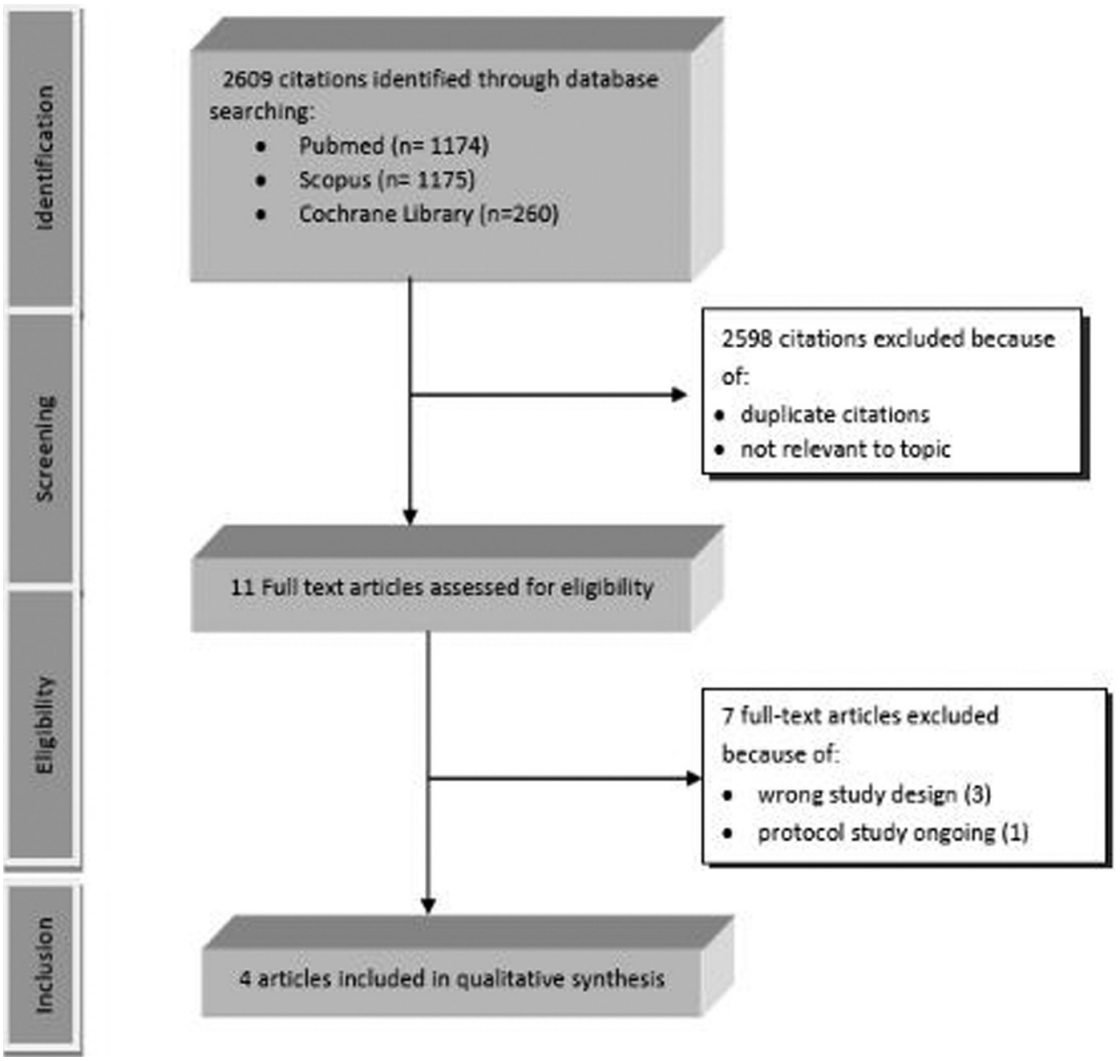
Prisma flowchart of study selection process.

The search strategy identified 2609 records from Medline, Scopus And Cochrane Library. After reading the titles and abstracts, 2598 studies were excluded, and 11 were selected. No article was identified in the manual search. After reading the full text of the 11 selected studies, 7 were excluded (10,16-21) and 4 of them met the inclusion criteria (22-25) (Fig. 1). A flowchart for the study selection process is presented in Figure 1.

-Study characteristics and heterogeneity

Of the 4 included studies, 2 randomized controlled trials (22,23) and 2 retrospective cohort studies (24,25) reported on 187 patients, which included 94 test subjects and 93 controls.

Two studies selected patients with generalized aggressive periodontitis (22,24), one study with localized aggressive periodontitis(25), and one study included Aggregatibacter actinomycetemcomitans-associated moderate to advanced periodontitis including both chronic and aggressive periodontitis and participants aged up to 70 years (23). Smokers were not excluded from studies except for one study who was also restricted to an African-American population (25).

Follow up times varied from 6 months to 15 months. However, not all studies reported 3 months results after initial therapy (22).

The mode of non-surgical treatment varied between full mouth instrumentation (22,23,25), or quadrant instrumentation in four visits within two weeks (24), and all participants of studies used 0.2% chlorhexidine mouthwashes after treatment during 10 to 15 days.

The dose and duration of antibiotic regimens used in the 4 included studies also varied, as outlined in Table 1,  Table 1 cont. Amoxicillin was administered in 375 and 500mg doses, while metronidazole was administered in 250 and 500mg doses. All doses were given three times daily, and the duration of antibiotic therapy ranged between 7 and 10 days.

**Table 1:**
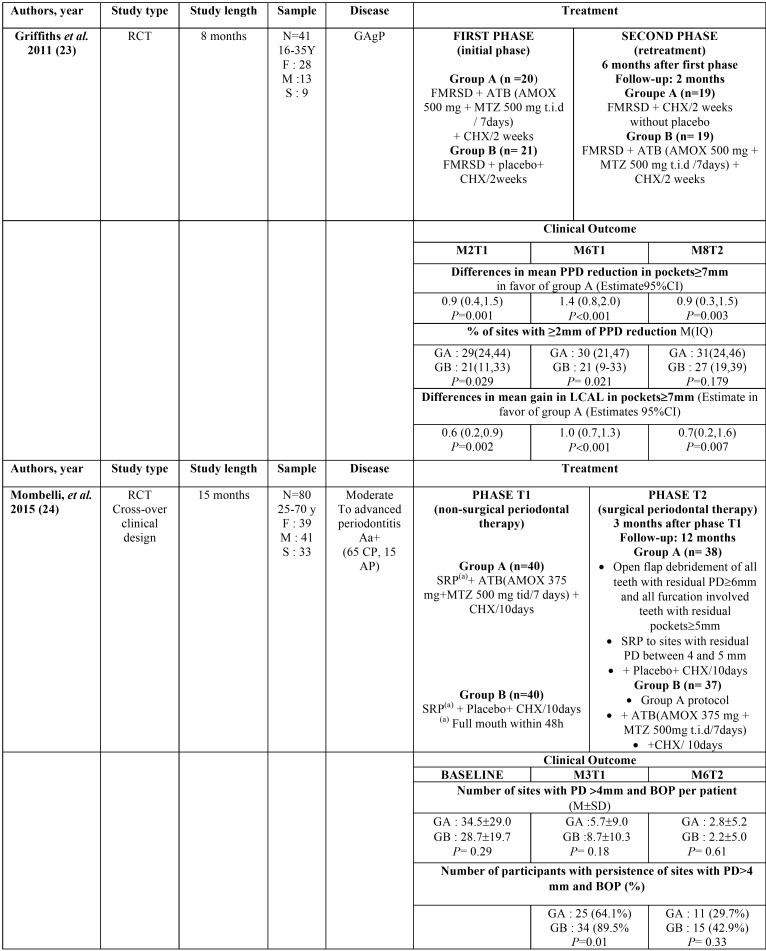
Summary of the characteristics and findings of the included studies.

**Table 1 cont.**
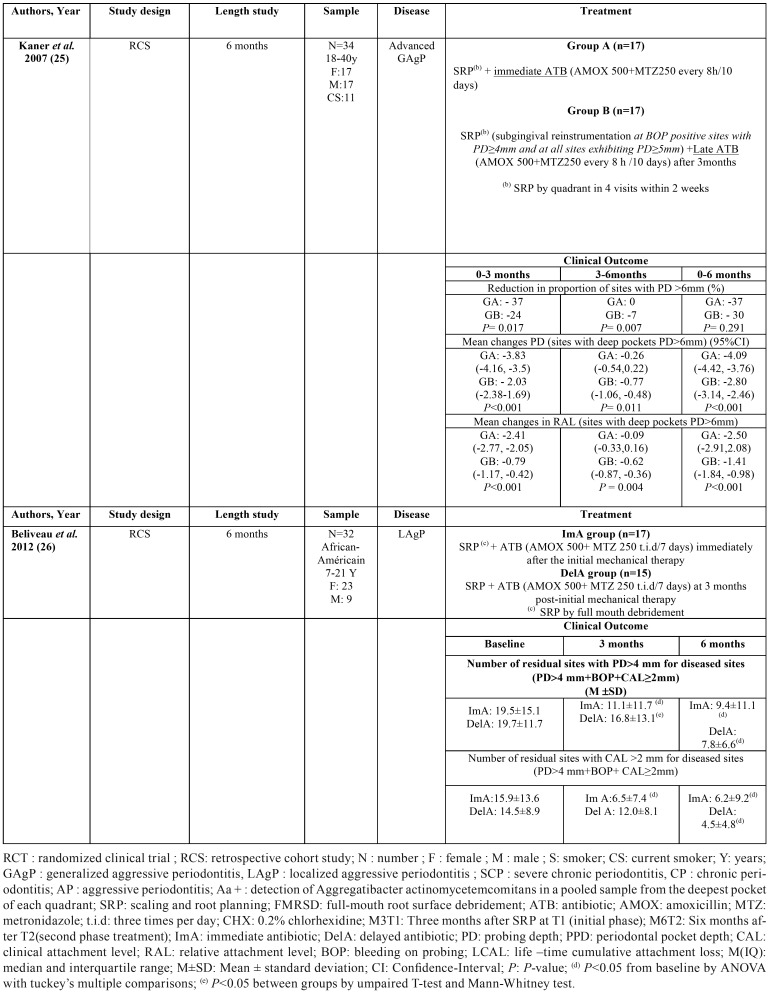
Summary of the characteristics and findings of the included studies.

A re-treatment was performed by all the studies in the reevaluation at 3 months after the initial phase (non-surgical treatment) except for one study in which the re-treatment was performed at 6 months after initial therapy (22).

Different approaches to treat the persistent residual pockets were tested at the re-evaluation phase consisting of a non-surgical re-treatment in all studies except for one study which performed both non-surgical and surgical re-treatment based on the values of the periodontal pockets depths, bleeding on probing and furcation involvement (23).

The primary outcomes studied were also heterogeneous between the included studies (PPD reduction at sites with initial PPD≥7mm (22), number of sites per patient with PPD>4mm and that bleed after probing (BOP+) (23), reductions of full mouth Probing Depth (PD) and Relative Attachment Levels (RAL)(24), reductions in mean Clinical Attachment Level (CAL) (25).

A summary of the characteristics of the included studies is reported in Table 1,  Table 1 cont..

-Results of Individual Studies:

In Griffith’s study (22), both treatments i.e. antibiotics initially and antibiotics at the re-treatment resulted in improvements of the mean PPD and LCAL(lifetime clinical attachment level) at both 6 and 8 months. The difference in mean PPD in deep pockets (≥7 mm) was statistically and clinically significant between the 2 groups, with a difference of 0.9mm (*P*<0.001) at 2 months, 1.4mm (*P*<0.001) at 6 months and 0.9mm (*P*<0.003) at 8 months in favor of the group who had antibiotic initially. Similar patterns were observed for LCAL with statistically significant differences, but with a lower greatness of difference.

According to the study of Mombelli (23), the administration of the amoxicillin plus metronidazole in the first or second phase resulted in similar long-term outcome. At 3 and 6 months after initial therapy, the mean number of sites with pocket depths PD >4 mm and BOP per patient was significantly lower than at baseline in both groups, with no significant difference between the two groups (*P*=0.18, *P*=0.61 respectively). However, administrating adjunctive antibiotics in the first phase result in fewer patient requiring further therapy, a shorter treatment time and a lower mean number of surgical interventions.

In the retrospective study of Kaner (24), giving systemic antibiotics as an adjunct to SRP resulted in significant clinical improvements over 6 months in both group A (immediate antibiotics group) and group B (late antibiotics group). However, except for BOP, the changes in all clinical parameters over time were significantly different between the two groups. At 3 months after the initial therapy, the immediate administration of antibiotics resulted in significantly greater reductions in PD and RAL (for full- mouth means and for initially deep sites) and in the proportion of sites with PD >6mm compared to SRP alone in the late antibiotics group. After 6 months, both groups showed comparable changes in full-mouth PD and RAL, BOP and pus, and the proportions of deep sites (PD>6mm). However, the immediate antibiotic group obtained statistically significantly higher reductions in the mean PD (-4.09 mm) and RAL (-2.50 mm) in deep sites (with PD>6 mm) in comparison with the late antibiotic group (-2.80 mm and -1.41 mm, respectively) (*P*<0.05).

The study of Beliveau (25) showed that administration of an antibiotic protocol immediately following mechanical therapy resulted in significant reductions in mean CAL at both 3 and 6 months post-initial therapy while a significant reduction was observed only at 6 months in the delayed antibiotic (DelA) group.

In addition, the percentage of sites with a PD >4mm was reduced in both immediate antibiotic (ImA) and DelA groups six months post-therapy, although a statistically significant reduction in PD three months following treatment was observed only in the ImA group (*P*<0.05).

A decrease in the percentage of sites with CAL>2mm at both 3 and 6 months was also observed in the ImA group, while this decrease was observed only at 6 months in the DelA group. Therefore, the early administration of antibiotics seems to result in a more rapid amelioration and maintenance of the major clinical parameters.

A summary of the results of the included studies is reported in Table 1.

-Risk of Bias Within Individual Studies:

The quality of each included clinical trial was assessed using the revised risk of bias assessment tool from the Cochrane Collaboration’s handbook version 5.1.0 (14). Based on the seven criteria analyzed, the Griffith’s study was estimated of “high risk of bias” and the Mombelli’s study “unclear risk of bias”., as it is depicted in Table 2.

**Table 2:**
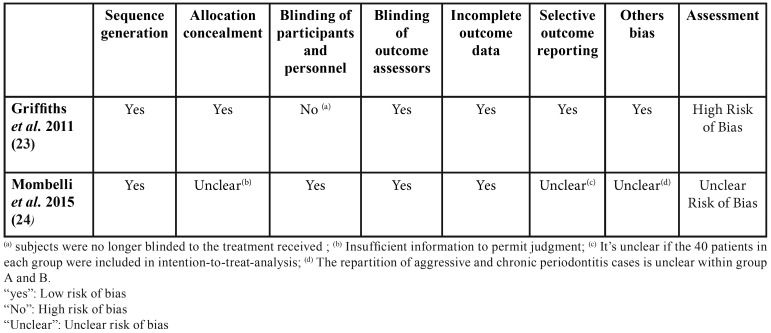
Assessment of risk of bias in randomized clinical trials (RCT) included in accordance with the Cochrane Handbook for Systematic Reviews of Interventions (14).

Even if classified at low risk of bias in six of the seven criteria analyzed, the Griffith’s study was estimated at high risk of bias because of the absence of blinding of the participants and personnel to the treatment received. The Mombelli’s study was classified as having unclear risk of bias based on the uncertainty around three sources of bias.

The quality of each retrospective cohort study included was assessed in accordance with the Newcastle-Ottawa quality assessment scale for cohort studies criteria and depicted in Table 3. The 2 RCS included scored individually 8 stars (4, 2 and 2 stars in respectively “selection”, “comparability” and “outcome” domains) and were rated good quality.

**Table 3:**
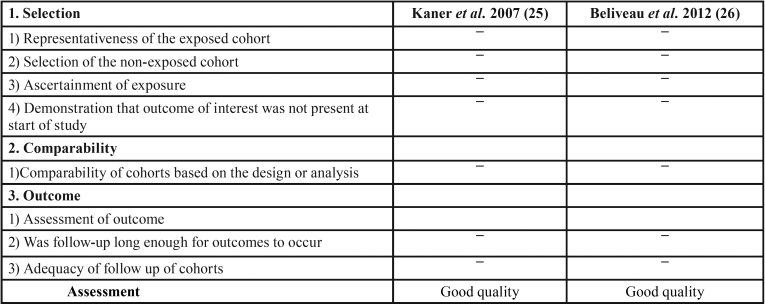
Assessment of risk of bias in retrospective cohort studies (RCS) included in accordance with the Newcastle-Ottawa quality assessment scale for cohort studies (15).

## Discussion

The results of this systematic review demonstrated that up until today, only 4 studies including 2 RCS (24,25) and 2 RCT (22,23) in the literature compared the clinical effects of systemic antibiotics administered at different phases of mechanical therapy.

Among them, 2 studies including 1 RCT (22) of high risk of bias and 1 RCS (24) of good quality suggested there were greater clinical benefits when metronidazole and amoxicillin association was administered during the active phase of periodontal therapy than in the non-surgical re-treatment phase. Individuals treated with antibiotics together with SRP in the initial phase of treatment, showed greater reduction in PD and greater gain in CAL in deep pockets at all the time points than individuals treated lately with antibiotics 3 or 6 months after SRP.

The 2 others studies including 1 RCT (23) of unclear risk of bias and 1 RCS (25) of good quality, included in this systematic review showed that giving antibiotics in the first or second phase have had no influence on the long term outcome. Six or 12 months after therapy, the mean number of sites with PD >4 mm and BOP per patient was significantly lower than at baseline in both groups, with no significant difference between the two groups. However, fewer patients treated with adjunctive antibiotics in the first phase needed further therapy (23,25), the treatment time in the second phase was shorter, and the mean number of surgical interventions was lower (23).

Furthermore, the differential microbiologic outcomes of this latest RCT was reported more recently and showed that giving the antibiotics during the first or second phase yielded similar microbiologic outcomes after 1 year although antibiotics in the first phase reduced bacterial counts quicker at 3 months (16). These findings were therefore in accordance with the clinical outcomes of this RCT (23).

It’s important to point out therefore that in all these 4 studies (22-25), the immediate antibiotic usage at initial therapy provided beneficial clinical responses earlier in the treatment compared to the its later usage at reevaluation phase, thereby reducing the need for further therapy, which is both surgical and non-surgical in the RCT of Mombelli (23).

It is also worth considering that these studies present some limitations. First, the Griffith’s study (22) was not initially designed to compare the effectiveness of the antibiotic given in different phases of treatment. It was a follow-up study of a randomized controlled trial which was performed in a previous study (26) in order to assess the adjunctive clinical effect of the administration of systemic amoxicillin and metronidazole in the non-surgical treatment of generalized aggressive periodontitis (GAP).

Second, in this study, no placebo has been used in the second phase and the longitudinal evaluation time was short. The different follow-up times between immediate antibiotic group and delayed antibiotic group (6 months from initial antibiotic versus 2 months respectively) would constitute an additional limitation.

Furthermore, the heterogeneity between studies regarding the study design, the populations studied, the type of disease and severity, the modalities of non-surgical therapy (full mouth debridement or quadrant debridement), dosage and duration of antibiotherapy, the approaches of re-treatment (non-surgical exclusively or both non-surgical and surgical treatment) and the primary and secondary outcomes, makes terminating conclusions about the best time to prescribe adjunctive antibiotics in periodontal therapy difficult even if it emerges through these studies an early benefit for antibiotic administration in the active phase of treatment.

The conclusion of this review is somewhat consistent with another previous systematic review that compared the clinical effectiveness of systemic antibiotics administered in the active stage of periodontal treatment or after the healing phase (8). Only one study (22) was included in this review which did not allow authors to draw any clear conclusion. The present systematic review has an added value from methodological point of view (PRISMA recommendations, information sources, assessment of risk of bias,..), leading to more included studies, recent and available evidence to address the research question.

One double-blinded, three armed, placebo-controlled and bi-centric RCT was also designed to address the question of which is the best time for the administration of antibiotics in patients with severe periodontitis but it’s still an ongoing trial and its findings have not been published yet (17).

Some biologic concepts support the use of antibiotics during the active phase of therapy in association with mechanical debridement (11). Indeed, early intake of antibiotics could result in rapid and significantly reduce in keystone pathogens leading to a greater recolonization of the periodontal pockets by beneficial species (27,28). Furthermore, the degree of periodontal tissue inflammation during the active phase of therapy, may provide a higher antibiotic concentration and uptake in subgingival space (24).

Concerning the clinical benefits of early antibiotic administration, it could reduce the need for subsequent non-surgical and surgical interventions because of a decreased number of residual pockets (29-33) which may represent an important benefit to patients in many terms. First, it may avoid the risk of hard tissue trauma from repeated attempts of instrumentation in locally unresponsive sites or sites with recurrent disease (34). Second, it may also avoid the risks, stress and financial costs associated with surgical procedures. Furthermore, the immediate antibiotic usage at initial therapy provided beneficial clinical responses earlier in the treatment compared to its later usage at reevaluation phase. This rapid suppression of the periodontal infection could avoid the higher risk of future disease progression and of the potential systemic health consequences (11).

On the other hand, delaying antibiotic therapy to the second surgical treatment phase may be supported by the fact that scaling and root planing alone can be enough to control disease progression in most cases of periodontitis. Furthermore, given the limitations of non-surgical mechanical debridement and the limited effects of antibiotics on intact biofilm, a pocket access surgery may be required to completely eliminate the subgingival biofilm and calculus and therefore potentiate the effectiveness of systemic antibiotic at re-treatment (35,36).

However, even though these arguments seem to be biologically plausible, the literature data do not support them (2).

Otherwise, the major benefit for the delay in prescribing the antibiotics at the reevaluation phase relates to the avoidance of the risks associated with the administration of antibiotics, such as the development of side effects and the emergence of ‘new’ antibiotic-resistant species (11).

Nonetheless, no major side effects are likely to be associated with the systemic administration of metronidazole and amoxicillin (26,29,31,37,38) and the potential risk of causing resistant strains against the antibiotics used in the periodontal therapy is a controversial issue in the current literature (39,40). It has been suggested that the increase in proportions of antibiotic resistant species in the subgingival biofilm seem to be rather the consequence of selection of organisms that were initially resistant to the antibiotic before antibiotic intake for periodontal purposes (41-43). Data assessing the emergence of ‘new’ antibiotic-resistant species are not available in the literature and conducting studies specifically designed to answer this question is very difficult (11).

Otherwise, according to Mombelli (36), the treatment of a periodontal infection, once in a punctual way, with two drugs with different antimicrobial action (Amoxicillin and Metronidazole), in a controlled situation following mechanical debridement, contributes probably little to the problem of resistance to antibiotics compared to the effects of the frequent prescription of antibiotics by dentists or doctors for other therapeutic and prophylactic purposes.

Therefore, in view of all these arguments, the recommendation to prescribe antibiotics in the active phase or at reevaluation in the treatment of periodontal infection should adhere to the same therapeutic principle used for the treatment of any other infection in the body, that is: the risks have to be broadly counterbalanced by expected benefits to the patient (11). In this regard, the possible negative effects of antibiotics including the potential risk of emergence of ‘new’ antibiotic-resistant species have to be balanced against the risk of delaying antibiotic prescription in terms of potential risk of systemic health consequences of a persisting periodontal infection, and the inconvenience, discomfort and financial consequences of further surgical or no-surgical retreatment (36).

Consequently, in our opinion, it may be judicious in the light of the new classification of periodontal diseases (44), to prescribe antibiotic as part of initial therapy in cases of periodontitis grade C, i.e. in cases where there is a risk of a rapid progression or a negative impact on systemic health and post-pone their prescription in the other cases of periodontitis at the reevaluation phase when mechanical debridement alone has not given successful results. However, our suggestion must be supported by more appropriate randomized clinical studies specifically designed for this purpose.

In conclusion, this systematic review didn’t find a strong evidence to determine with certainty the ideal time for the adjunctive use of antibiotics. Some of included studies in this systematic review were of questionable quality of evidence. Furthermore, their heterogeneity makes comparison difficult and terminating conclusions even more. Despite this insufficient evidence, it seems that prescription of systemic antibiotic at the active phase of periodontal therapy leads to better clinical outcomes.

In future, more appropriate and standardized randomized clinical trials with longer follow-up periods must be conducted and completed by microbiological and immunological studies in order to evaluate the impact of timing of adjunctive antibiotic therapy on the outcome of periodontal therapy. Additional research into the pharmacokinetics of antibiotics in periodontal tissues during the different phases of periodontal therapy is also required to get a complete picture of the problem addressed.
